# The DNA damage response in mammalian oocytes

**DOI:** 10.3389/fgene.2013.00117

**Published:** 2013-06-24

**Authors:** John Carroll, Petros Marangos

**Affiliations:** ^1^School of Biomedical Sciences, Faculty of Medicine, Nursing and Health Sciences, Monash UniversityMelbourne, VIC, Australia; ^2^Department of Biological Applications and Technology, University of IoanninaIoannina, Greece; ^3^Department of Cell and Developmental Biology, University College LondonLondon, UK

**Keywords:** oocytes, DNA damage response, meiotic recombination, p63, DNA damage checkpoint, meiosis, prophase arrest, apoptosis

## Abstract

DNA damage is one of the most common insults that challenge all cells. To cope, an elaborate molecular and cellular response has evolved to sense, respond to and correct the damage. This allows the maintenance of DNA fidelity essential for normal cell viability and the prevention of genomic instability that can lead to tumor formation. In the context of oocytes, the impact of DNA damage is not one of tumor formation but of the maintenance of fertility. Mammalian oocytes are particularly vulnerable to DNA damage because physiologically they may lie dormant in the ovary for many years (>40 in humans) until they receive the stimulus to grow and acquire the competence to become fertilized. The implication of this is that in some organisms, such as humans, oocytes face the danger of cumulative genetic damage for decades. Thus, the ability to detect and repair DNA damage is essential to maintain the supply of oocytes necessary for reproduction. Therefore, failure to confront DNA damage in oocytes could cause serious anomalies in the embryo that may be propagated in the form of mutations to the next generation allowing the appearance of hereditary disease. Despite the potential impact of DNA damage on reproductive capacity and genetic fidelity of embryos, the mechanisms available to the oocyte for monitoring and repairing such insults have remained largely unexplored until recently. Here, we review the different aspects of the response to DNA damage in mammalian oocytes. Specifically, we address the oocyte DNA damage response from embryonic life to adulthood and throughout oocyte development.

## THE DNA DAMAGE RESPONSE

Cells respond to DNA damage created in the form of single strand breaks (SSBs) or double strand breaks (DSBs) by arresting their cell cycle to allow time for the damage to be repaired. Therefore, the DNA damage response (DDR) involves cell cycle arrest through the activation of DNA damage checkpoints (DDCs) and DNA damage repair mechanisms. The DDR sequence of events is tightly coordinated so that cell cycle arrest is lifted as soon as the damage has been repaired. When the extent of damage does not allow full repair, programed cell death mechanisms become active in order to remove, through apoptosis, the permanently damaged cells (Bartek and Lukas, 2007; Ciccia and Elledge, 2010).

**FIGURE 1:**
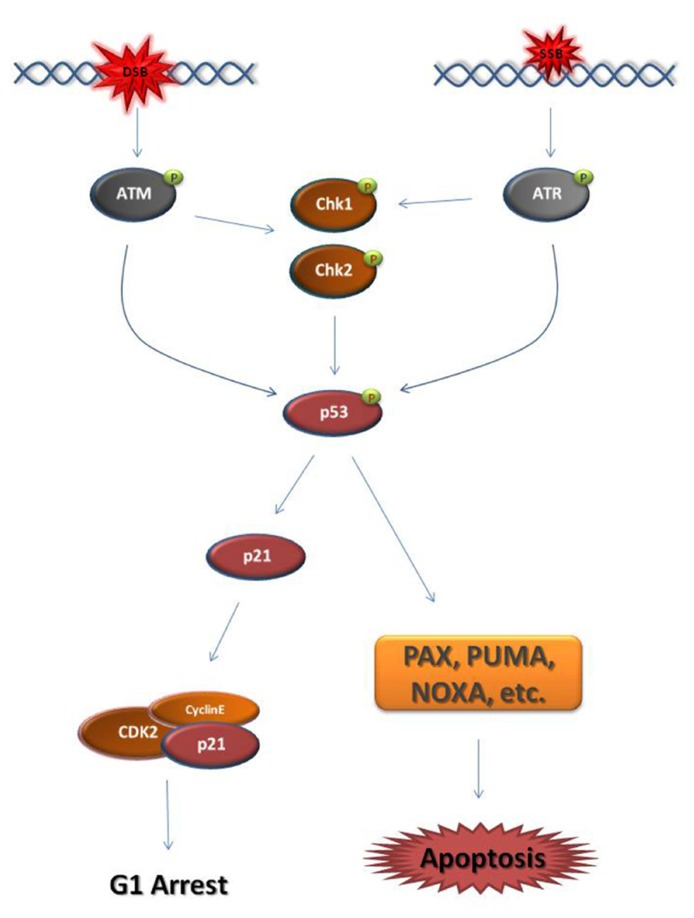
**Schematic representation of the major steps of the G1 DNA damage checkpoint**. Double strand breaks (DSB) and single strand breaks (SSB) cause the activation of the master DNA damage checkpoint kinases ATM and ATR, respectively. ATM phosphorylates and activates the downstream effector checkpoint kinases Chk1 and Chk2, while ATR activates Chk1. ATM/ATR and the checkpoint kinases activate the transcription factor p53. p53 drives the transcription of the cyclin-dependent kinase (CDK) inhibitor p21. p21 binds and inhibits CDKs responsible for progression into S-phase, such as Cyclin E-CDK2. As a result, the cell cycle arrests at G1. When the DNA damage cannot be repaired, p53 drives the cell to apoptosis through the transcription of pro-apoptotic genes, such as PAX, PUMA, and NOXA. It must be noted that this figure is an oversimplified representation of the pathways enabled in response to DNA damage at G1 phase. 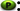 activating phosphate.

Eukaryotic cells activate DDR mechanisms primarily at the G1/S-phase transition and the G2/M-phase transition. In both cell cycle phases, DSB or SSB establish a DDC by triggering the activation of the master kinases ATM (ataxia telangiectasia mutated) and ATR (ataxia telangiectasia and Rad3-related), respectively (Reinhardt and Yaffe, 2009; Smith et al., 2010). At G1, the major downstream effector of the ATM/ATR kinases is the transcription factor p53, also known as “the guardian of the genome” (**Figure 1**; Kastan and Lim, 2000; Bartek et al., 2007). When activated, p53 blocks the transcription of cell cycle regulators that normally induce the G1/S-phase transition, such as cyclin E, while driving the transcription of factors that block the G1/S-phase transition, such as the cyclin-dependent kinase (CDK) inhibitor, p21 (Rocha et al., 2003; Mirzayans et al., 2012). p53 is also the primary inducer of apoptotic mechanisms following DNA damage (Fridman and Lowe, 2003; Meulmeester and Jochemsen, 2008).

**FIGURE 2:**
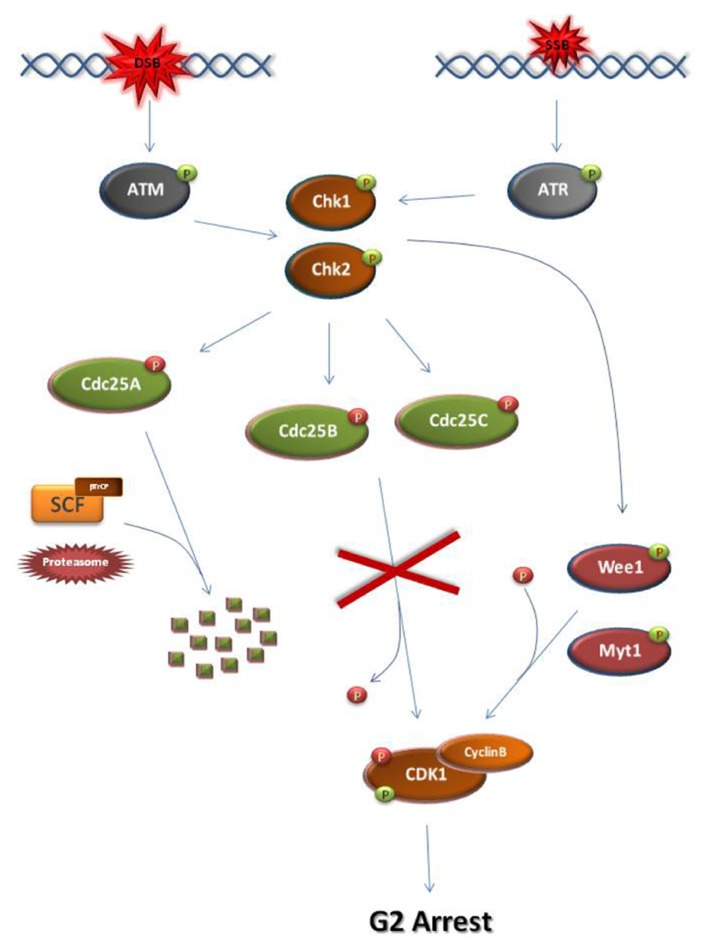
**Schematic representation of the major steps of the G2 DNA damage checkpoint**. At S/G2 phase, DSBs and SSBs activate ATM and ATR, respectively. As a consequence, checkpoint kinases Chk1 and Chk2 become activated. Chk1 and Chk2 can directly phosphorylate and activate Wee1 or Myt1 kinases. Wee1/Myt1 impose inhibitory phosphorylations on the M-phase kinase Cyclin B-CDK1 in order to block M-phase entry. At the same time, the checkpoint kinases directly phosphorylate and inhibit Cdc25 phosphatases. Unlike the inhibitory phosphorylations of Cdc25B and Cdc25C, checkpoint kinase-dependent phosphorylation of Cdc25A allows its recognition by the SCF/βTrCP ligase. Subsequent ubiquitination of Cdc25A renders the phosphatase a substrate for the proteasome leading to its degradation. As a result of their inhibition, the Cdc25 phosphatases cannot remove the inhibitory phosphate from CDK1. Consequently, the cell cycle arrests at G2 due to inhibition of CDK1 activation. The activating CDK1 phosphorylation is introduced by CDK-activating kinases but is masked by the Wee1/Myt1 inhibitory phosphorylations. 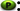 activating phosphate. 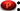 inhibiting phosphate.

At G2, establishment of the DDC and subsequent M-phase entry inhibition requires the ATM/ATR-dependent activation of checkpoint kinases, Chk1 and Chk2 (**Figure 2**; Bartek and Lukas, 2007; Smith et al., 2010). Normally, entry into M-phase is obtained by the activation of the universal M-phase regulator, cyclin B-CDK1 (Doree and Hunt, 2002; Lindqvist et al., 2009). Cyclin B-CDK1 activation requires cyclin B synthesis and the activation of Cdc25 phosphatases which lift CDK1 inhibitory phosphorylations established by CDK1 inhibitors such as Wee1 and Myt1 kinases (Aressy and Ducommun, 2008; Potapova et al., 2009). Following DNA damage at G2, Chk1/Chk2 kinases cause the inhibition of cyclin B-CDK1 activation by disrupting the action of Cdc25 either through facilitating SCF (Skp, Cullin, F-box) ligase-dependent degradation, as in the case of Cdc25A or through inhibitory phosphorylation (Cdc25B, Cdc25C; Mailand et al., 2000; Busino et al., 2003; Ferguson et al., 2005; Boutros et al., 2007).

During the DDC-mediated arrest, DNA damage is repaired by a number of different mechanisms depending on the nature of the damage. Single strand damage is repaired by three main repair pathways: base excision repair (BER), nucleotide excision repair (NER), and mismatch repair (MMR). Two are the major mechanisms involved in DSB repair, namely homologous recombination (HR) and non-homologous end joining (NHEJ; Aguilera and Gomez-Gonzalez, 2008; Cohn and D’Andrea, 2008).

## PROPHASE ARREST

The oocyte is a unique cell that differs significantly both from somatic cells but also from the male germ cells in respect to its cell cycle, its functions and its purpose. A unique characteristic of the oocyte, not seen in any other cell type, is prophase arrest.

**FIGURE 3:**
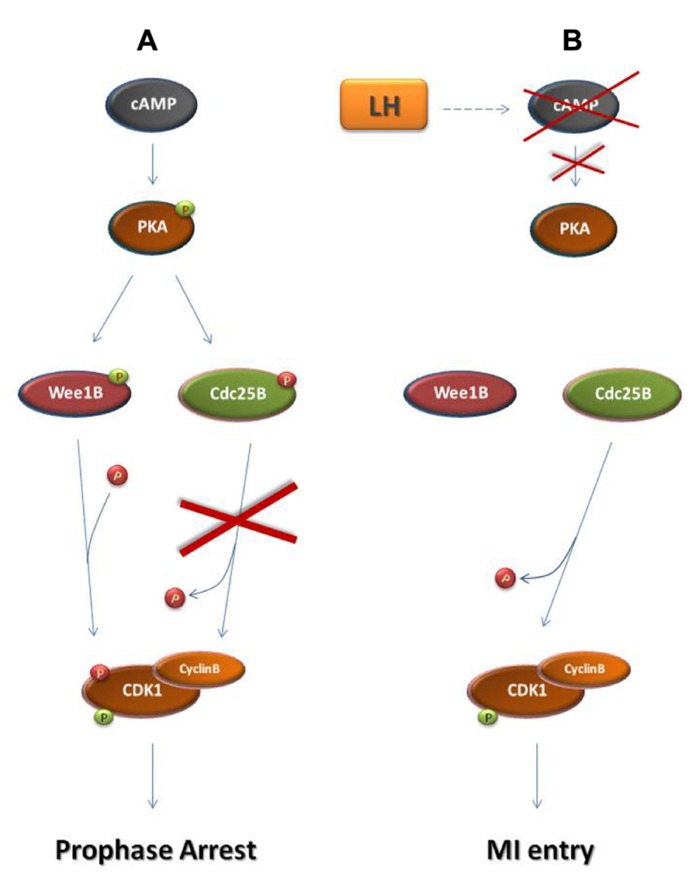
**Regulation of prophase arrest and resumption of meiosis**. **(A)** Regulation of prophase arrest. During the arrested state, a signaling pathway that is established in response to the interactions of the oocyte with its neighboring granulosa cells leads to the accumulation of cAMP in the oocyte. cAMP causes the phosphorylation of protein kinase A (PKA). Similarly to Chk1 and Chk2 during the G2 DNA damage checkpoint, PKA in prophase-arrested oocytes phosphorylates and activates Wee1 kinase and specifically the Wee1 isoform Wee1B. Furthermore, PKA phosphorylates and inhibits Cdc25B phosphatase. As a result, Cyclin B-CDK1 remains inactive and the oocyte arrested in meiotic prophase. **(B)** Resumption of meiosis. During the estrus cycle, the surge of the luteinizing hormone (LH) drives a signaling cascade that results in the decline of cAMP levels in the oocyte. This leads to PKA inactivation, ending the PKA-dependent phosphorylation of Wee1B and Cdc25B. Just as in M-phase entry in somatic cells, Wee1B becomes inactive and Cdc25B is activated in order to remove the inhibitory phosphates from CDK1. In response to Cyclin B-CDK1 activation, the oocyte enters the first meiotic M-phase. 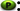 activating phosphate. 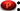 inhibiting phosphate.

The mechanisms regulating meiotic prophase arrest and resumption of meiosis resemble the establishment of the somatic cell G2 DDC and checkpoint recovery, respectively (**Figures 2 and 3**; Bassermann et al., 2008; Solc et al., 2010). The major common element in both systems is the alteration of cyclin B-CDK1 activity, predominantly through the action of CDK1 activators and inhibitors (Bassermann et al., 2008; Solc et al., 2010).

Before the end of gestation oocytes become arrested at the dictyate stage of the meiotic prophase (Rodrigues et al., 2008). During prophase arrest in oocytes, cyclin B-CDK1 remains inactive due to the maintenance of high levels of cAMP within the oocyte and the subsequent sustained activation of protein kinase A (PKA; **Figure 3**; Mehlmann et al., 2002; Schmitt and Nebreda, 2002). PKA phosphorylates and inactivates the Cdc25 isoform Cdc25B which is responsible for cyclin B-CDK1 activation in oocytes (Lincoln et al., 2002; Pirino et al., 2009; Oh et al., 2010). Furthermore, PKA phosphorylates and activates the CDK1 inhibitor Wee1B which is the oocyte-specific Wee1 isoform (Han et al., 2005; Oh et al., 2010). Following the rise in the levels of luteinizing hormone (LH), during the estrus cycle, cAMP levels drop and PKA becomes inactive allowing CDC25B activation and the subsequent cyclin B-CDK1 activation leading to entry into the first meiotic M-phase (MI; Lincoln et al., 2002; Marangos and Carroll, 2004; Solc et al., 2010).

Most of the information we possess regarding the mammalian oocyte DDR involves prophase arrest.

## MEIOTIC RECOMBINATION CHECKPOINTS

In mammalian oocytes, DNA breaks are first identified during meiotic recombination. Meiotic recombination is a process that takes place before birth from the leptotene to the pachytene stage of meiotic prophase and involves the natural formation of DSBs. Since meiotic recombination processes are extensively reviewed elsewhere (Lydall et al., 1996; Roeder and Bailis, 2000; Burgoyne et al., 2009; Martinez-Perez and Colaiacovo, 2009; Kurahashi et al., 2012) we will limit our analysis to a very general overview of the major aspects of the recombination-induced DDR in mammalian oocytes.

At the start of meiotic prophase (embryonic day 13–18.5 post-coitus in female mice), homologous chromosomes pair along their full length in a process called synapsis (Roeder, 1997; Livera et al., 2008; Martinez-Perez and Colaiacovo, 2009). During synapsis and following the initiation of homolog pairing, DNA DSBs appear within the chromosomes. These DSBs allow DNA exchange between homologous non-sister chromatids through genetic recombination (McDougall et al., 2005; Burgoyne et al., 2009). Recombination leads to the formation of natural bridges, called chiasmata, which hold the homologous chromosomes together until MI allowing their attachment from the opposite poles of the MI spindle and their alignment at the metaphase I plate (McDougall et al., 2005; Burgoyne et al., 2009). Therefore, formation of chiasmata during meiotic recombination ensures the correct segregation of homologous chromosomes during the first meiotic division. Meiotic recombination dysfunctions can cause damaged genomes and the formation of aneuploid gametes (Burgoyne et al., 2009; Yanowitz, 2010; Kurahashi et al., 2012). Therefore, meiotic cells have developed checkpoint mechanisms around the pachytene stage of meiotic prophase in order to ensure the integrity and the completion of recombination (Lydall et al., 1996; Roeder and Bailis, 2000). In mammals, the activation of the recombination pachytene checkpoint when meiotic cells do not complete HR in time leads to cell death through apoptosis (Lydall et al., 1996; Roeder and Bailis, 2000). In oocytes, dysfunction of factors involved in the recombination process leads to apoptosis at the perinatal period (Pittman et al., 1998; Baudat et al., 2000; Di et al., 2005).

In meiotic recombination, it is well established that Spo11 is the main factor to promote the formation of DSBs (Baudat et al., 2000; Di et al., 2005). However, recent findings have shown that homolog pairing is completely abolished in Spo11^-^^/^^-^ spermatocytes suggesting that Spo11 is also required for the DSB-independent initiation of synapsis (Boateng et al., 2013). Absence of the Spo11-dependent homolog pairing and DSB formation leads to oocyte apoptosis during early follicular development, soon after birth (Baudat et al., 2000; Di et al., 2005). Similar observations are made in mice lacking Spo11-associated proteins, such as Mei4 (Kumar et al., 2010). In Spo11 null mice, the oocytes that survive and acquire the competence to enter M-phase cannot segregate their homologous chromosomes properly due to the absence of chiasmata and remain arrested at MI (Cole et al., 2010).

Other factors, such as ATM and DMC1 are responsible for rejoining the DNA strands (Pittman et al., 1998; Yoshida et al., 1998; Roeder and Bailis, 2000; Di et al., 2005). Besides its role in DDC establishment, ATM is a crucial component of HR repair mechanisms (Smith et al., 2010). The importance of these proteins in DNA strand rejoining is shown by the fact that the absence of DMC1 or ATM leads to programed cell death in prophase oocytes and DMC1 null and ATM null mice are infertile as are Spo11 null mice (Pittman et al., 1998; Yoshida et al., 1998; Baudat et al., 2000; Di et al., 2005). However, in the case of DMC1 and ATM, the oocytes do not reach the stage of becoming enclosed in follicles and degenerate, through apoptosis earlier than Spo11 null oocytes (Pittman et al., 1998; Yoshida et al., 1998; Baudat et al., 2000; Di et al., 2005). Furthermore, in DMC1-Spo11 and ATM-Spo11 double mutants the oocyte reserve depletion phenotype resembles the one seen in Spo11 null mice, which leads to the conclusion that the Spo11 mutation is epistatic to the DMC1 and ATM mutations (Di et al., 2005). These results indicate that, unlike Spo11 mutants, the different phenotype of the DMC1 and ATM mutants is possibly the result of persistent, unrepaired DNA damage.

Besides ATM, other traditional ATM-dependent DDR factors are activated at the sites of meiotic recombination-induced DNA damage in order to amplify the DSB signal, such as ATR kinase, BRCA1 and the phosphorylated form of the nucleosomal histone H2AX (γH2AX; Xu et al., 2003; Burgoyne et al., 2007). However, in the absence of DSBs, ATR, BRCA1, and γH2AX are recruited on unsynapsed homologous chromosomes in order to impose their transcriptional silencing (Turner et al., 2005; Mahadevaiah et al., 2008; Burgoyne et al., 2009). If synapsis is not successful, transcriptional silencing can lead to apoptosis if important active genes cease to function (Burgoyne et al., 2009; Kurahashi et al., 2012). This DSB-independent process allows the elimination of oocytes with unsynapsed chromosomes and could explain the Spo11^-^^/^^-^ oocyte death phenotype.

Therefore, there appear to be two checkpoint responses to recombination defects in oocytes: a DNA DSB-dependent response triggered by unrepaired DSBs and a DNA DSB-independent response triggered by the absence of synapsis. In both cases, the activation of the checkpoint will lead to apoptosis. However, it is not yet determined how unrepaired, recombination-induced DSBs would trigger apoptosis in oocytes.

## p63-DEPENDENT PATHWAY

In mammalian oocytes, DSBs induced as a consequence of genotoxic stress trigger the activation of a TAp63-dependent mechanism which drives affected oocytes to apoptosis (Suh et al., 2006; Kerr et al., 2012a).

TAp63 is an isoform of p63 which belongs to the p53 family of transcription factors. This protein family includes three transcription factors, namely p53, p63, and p73 (Levine et al., 2011). Besides being important for the activation of DDR mechanisms, mainly cell cycle arrest and apoptosis of damaged cells, these factors also possess a wide range of other functions including their involvement in maternal reproductive efficiency. p53 has been shown to regulate embryo implantation (Hu et al., 2007). TAp73, a p73 isoform, is involved in the M-phase spindle assembly checkpoint and mice lacking TAp73 are infertile. In female TAp73^-^^/^^-^ mice, infertility is due to chromosome missegregation leading to chromosomal abnormalities in the dividing oocyte and pre-implantation stage embryo (Tomasini et al., 2008; Levine et al., 2011). TAp63, a p63 isoform, is the only p53 family member identified so far to participate in the oocyte DDR. Although, TAp63 is not expressed in the male germ cells, a newly identified hominidae isoform, GTAp63, seems to possess DDR functions in males (Beyer et al., 2011; Amelio et al., 2012).

**FIGURE 4:**
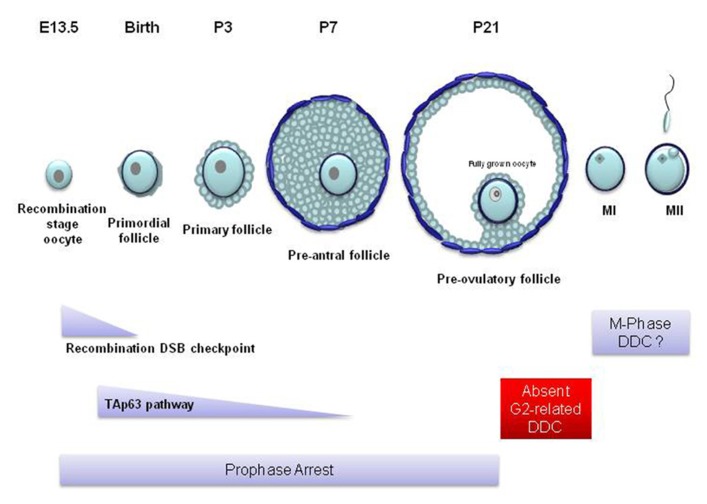
**Mammalian oocyte DNA damage checkpoints in relation to follicular and oocyte development**. In most mammals, only a few hundred oocytes reach the competence to become fertilized. At the beginning of oogenesis, mitotically dividing oogonia proliferate to form a population of a few million. Most become destroyed through apoptosis while all the remaining enter meiosis before birth. These oocytes become surrounded by a single layer of epithelial cells forming primordial follicles. Following birth, ovarian follicles from this primordial pool mature spontaneously into primary and secondary follicles. During this stage of follicular maturation the oocyte grows in size and becomes surrounded by more layers of proliferating follicular cells which are in turn surrounded by layers of theca cells. However, these pre-antral follicles never reach full maturity and soon become atretic and deteriorate. At puberty and following the rise in the levels of the follicle stimulating hormone (FSH), during every estrus cycle, a small number of follicles mature beyond the pre-antral stage forming an antrum (antral follicle). From these follicles only a few reach the pre-ovulatory stage (Rodrigues et al., 2008). In the mouse, sustained unrepaired recombination-induced DSBs trigger oocyte apoptosis following the pachytene stage of meiotic prophase during embryonic life. These oocytes rarely survive to form primordial follicles around the time of birth. Genotoxic stress activates TAp63-dependent apoptosis at the diplotene stage of prophase. TAp63-induced apoptosis affects oocytes from the primordial stage of follicle development up to the pre-antral stage (primary and secondary follicles). Apoptosis does not appear in later stages of oocyte development. From the large pre-antral to the pre-ovulatory follicular stage, the oocyte remains in prophase arrest which may allow any inflicted DNA damage to be repaired. The fully grown oocyte which possesses the competence to resume meiosis and enter the first meiotic M-phase (MI) cannot establish cell cycle arrest checkpoints in response to DNA damage. It is possible that such checkpoints may be activated during meiotic M-phase. E, embryonic day; P, postnatal day; MII, meiotic M-phase II.

TAp63 is found in the nucleus of oocytes enclosed in primordial, primary and early pre-antral follicles (**Figure 4**) but is completely lost in the more mature, antral, follicles (Suh et al., 2006). TAp63 expression begins at embryonic day 18.5 up to adulthood (Suh et al., 2006; Livera et al., 2008). p63 has also been found in human embryonic stage oocytes (Livera et al., 2008). Nevertheless, TAp63 seems to be completely dispensable for oogenesis and the loss of TAp63 does not affect the oocyte reserve. The importance of TAp63 for the oocyte DDR was first identified in TAp63 null mice. In wild type and p53^-^^/^^-^ animals, ionizing radiation causes the complete deterioration and loss of primordial follicles, while the larger pre-antral follicles remain unaffected. However, the oocytes in primordial follicles of the TAp63 null mice were resistant to irradiation and cell death (Suh et al., 2006). These experiments showed that TAp63 induces cell death in primordial follicle oocytes with damaged DNA and that this function is not shared with p53. It must be noted that p63 seems to be only involved in DNA damage-dependent apoptosis since the rate of physiological embryonic oocyte death in p63^-^^/^^-^ ovaries is not different from wild type ovaries (Livera et al., 2008).

It has been proposed that, following DNA damage in oocytes, TAp63 becomes activated through phosphorylation by c-Abl tyrosine kinase (Gonfloni et al., 2009). Gonfloni and colleagues have shown that c-Abl inhibition by imatinib or GNF-2 protected oocytes from apoptosis in response to cisplatin-induced DNA damage (Gonfloni et al., 2009; Maiani et al., 2012). p63 phosphorylation drives resting inactive dimmers to form tetramers which possess the ability to bind DNA and activate the transcription machinery (Deutsch et al., 2011). The possible mechanism for Tap63 activation could involve the DNA damage-induced activation of the stress kinase c-Jun N-terminal kinase (JNK). In somatic cells, JNK phosphorylates the 14-3-3 proteins which under physiological conditions sequester c-Abl in the cytoplasm (Yoshida et al., 2005). 14-3-3 phosphorylation releases c-Abl to become transported into the nucleus in order to phosphorylate and activate Tap63. However, there has been some skepticism regarding c-Abl involvement because pharmacological agents, such as imatinib have occasionally been unable to inhibit oocyte apoptosis (Kerr et al., 2012b; Maiani et al., 2012). At the moment, it appears that the controversy surrounding c-Abl would only be resolved conclusively by genetically removing c-Abl from the female germ line.

Another very important question, however, is: which are the transcriptional targets of Tap63 that trigger apoptosis? Recently, two such targets have been identified in mouse oocytes, namely PUMA and NOXA (Kerr et al., 2012c). Both proteins belong to the pro-apoptotic arm of the Bcl-2 family and they have been known to inhibit pro-survival Bcl-2 proteins and promote the function of BAX and BAK, two major pro-apoptotic Bcl-2 family members, which in turn enable the mitochondria-induced apoptosis mechanisms (Chipuk and Green, 2008; Youle and Strasser, 2008). TAp63 enables the transcription of both PUMA and NOXA in mouse primordial follicle oocytes. In addition, PUMA^-^^/^^-^ mice and especially the double mutants PUMA^-^^/^^-^ NOXA^-^^/^^-^ mice, do not lose their primordial follicle pool in response to genotoxic stress (Kerr et al., 2012c). Therefore, TAp63-dependent PUMA and NOXA expression is most possibly responsible for driving oocyte apoptosis following DNA damage.

The knock-out mouse models of TAp63, PUMA and NOXA have shown that inhibition of the TAp63 pathway can rescue the primordial follicle oocyte pool from apoptosis following DNA damage. These observations could open novel medical options in order to sustain the fertility of women undergoing cancer therapy. It is well known that chemotherapy and radiation therapy for treating cancer leads to depletion of the ovarian oocyte reserve and leads to premature ovarian failure (POF) and hence premature menopause (Maltaris et al., 2007). Therefore, possible treatments that are based on the inhibition of the TAp63 pathway could allow the preservation of the oocyte pool following cancer therapy. However, is it safe to allow damaged oocytes to survive following cancer treatment? It would be expected that these oocytes carry significant damage that could be transferred to their offspring. However, an exciting result refutes these concerns: although wild type mice lose their primordial follicle reserve and become infertile following genotoxic stress, PUMA^-^^/^^-^ and PUMA^-^^/^^-^ NOXA^-^^/^^-^ female mice exposed to ionizing radiation have viable, healthy, and fertile offspring at the same rate as wild type mice not exposed to DNA damage (Kerr et al., 2012c). This finding suggests that during their long prophase arrest, oocytes possess the ability to repair DNA damage efficiently. Although more work needs to be done before treatments are obtained, these observations bring hope to cancer patients facing infertility.

The fact that genotoxic stress does not prevent the preservation of healthy oocytes when the TAp63 pathway is inhibited, raises an interesting question: why is a TAp63-dependent apoptotic pathway needed in oocytes? The answer might lie slightly before TAp63 expression, at the time of meiotic recombination. Quite conveniently, TAp63 is expressed following the physiological recombination-induced DSB repair. Immunofluorescence experiments show that γH2AX foci representing recombination-induced DSBs do not co-exist with TAp63. In mouse oocytes, γH2AX staining disappears by E18.5 by which time TAp63 becomes apparent (Livera et al., 2008). In this way, oocytes undergoing meiotic recombination are not in danger of apoptosis. However, as previously discussed, sustained DSBs during recombination, trigger the establishment of oocyte death mechanisms. The activation of these processes during and following the pachytene stage of prophase coincides with the appearance of TAp63. Therefore, TAp63 may be the “guardian of meiotic recombination,” driving to apoptosis any oocytes that fail to rejoin their chromosome arms on time.

These observations support the hypothesis that the original role, in evolutionary terms, of TAp63 may be the protection of the gene pool from meiotic recombination failure and not necessarily from externally inflicted genotoxic insults. Therefore, the deleterious role of TAp63 following exogenous genotoxic stress might be an undesired remnant of a p63-related recombination checkpoint.

At the moment, it remains unknown what processes take place at the late pre-antral follicular stage that would lead to the disappearance of TAp63 and TAp63-dependent apoptosis. Nevertheless, it is a fact that a p63-dependent apoptotic mechanism is absent in antral follicles. Therefore, an important question that arises is how the non-apoptotic mature antral and pre-ovulatory follicles (**Figure 4**) respond to oocyte DNA damage.

## PROPHASE TO MI TRANSITION

From the antral and up to the pre-ovulatory follicle, the response to DNA damage may involve the activation of repair mechanisms alone. At these stages of follicular development, physiological prophase arrest means that a DNA damage-induced checkpoint is not required to halt the cell cycle in order to permit repair. However, the fully grown oocyte in the pre-ovulatory follicle would not be expected to respond to DNA damage solely by repair mechanisms, but also by cell cycle arrest checkpoints. At this stage, the oocyte has reached full cytoplasmic and nuclear development and has acquired the competence to enter meiotic M-phase as soon as the LH surge occurs (Eppig, 1996). In the mouse, cell cycle regulators that are important for M-phase entry, such as cyclin B, CDK1, and Cdc25 accumulate in the fully grown, pre-ovulatory oocyte (Kanatsu-Shinohara et al., 2000).

Considering the resemblance of meiotic prophase arrest and the G2 DDC, it would be anticipated that fully grown oocytes employ similar DDR mechanisms as the ones present in somatic cell G2 phase. Therefore, it is surprising that a DDC is not established in response to DNA damage in M-phase competent oocytes.

Studies in mouse oocytes have shown that radiation-induced DNA damage may cause chromosomal aberrations, such as aneuploidy, translocations, chromatid interchanges and breaks (Tease, 1983; Jacquet et al., 2005). Past studies hinted at the possibility of a limited DDR in fully grown oocytes (Mailhes et al., 1994; Bradshaw et al., 1995). More specifically, it has been shown that a significant delay in the duration of MI is not observed following injection into female mice of etoposide, a topoisomerase II inhibitor and DSB inducer (Mailhes et al., 1994; Bradshaw et al., 1995).

Recently, the fully grown oocyte DDR has been examined in greater detail. In fully grown oocytes, DNA damage in the form of DSBs, that would normally cause G2 arrest in somatic cells, does not affect the timing and rate of entry into M-phase (Marangos and Carroll, 2012). Although, a DDC is not being established efficiently, DNA damage detection is effective. This has been determined by the presence of γH2AX at the DSB sites. A DDC is only established following very severe DNA damage inflicted by high concentrations of Etoposide or the DNA intercalating agent Doxorubicin, causing a significant delay in M-phase entry (Marangos and Carroll, 2012). Similar observations were also seen with the use of another DSB-inducing agent Neocarzinostatin (Yuen et al., 2012). Nevertheless, even following severe DNA damage and prolonged arrest, oocytes will eventually enter M-phase. The failure to establish a DDC in prophase-arrested oocytes could be attributed to checkpoint adaptation: a mechanism, which in somatic cells, involves Polo-like kinase 1 (Plk1) and Claspin and leads to the eventual inactivation of the G2 DDC in the presence of irreversible DNA damage (Yoo et al., 2004; Syljuasen et al., 2006).

The molecular basis for the absence of a reliable DDC in response to DSBs appears to be due to a limited ability to activate ATM kinase (Marangos and Carroll, 2012). The lack of ATM activity also affects the activation levels of downstream effectors such as Chk1. Low levels of expression of ATM in fully grown oocytes could be the reason for limited ATM activity. Another possibility could be the distinct chromatin configuration in fully grown oocytes (Marangos and Carroll, 2012). The fully grown oocyte is subjected to chromatin histone modifications such as deacetylation and methylation which are crucial for chromatin condensation and transcriptionally inactive heterochromatin formation (Mattson and Albertini, 1990; De La Fuente, 2006; Ma et al., 2012). Considering that the DDR and ATM specifically are known to be influenced by changes in chromatin structure and chromatin condensation (Bakkenist and Kastan, 2003), one hypothesis might be that DDR mechanisms are either not able to engage or are not triggered due to the fully grown oocyte specialized chromatin configuration.

The induction of Cdc25A degradation and Cdc25B inactivation are also inhibited following DNA damage in fully grown oocytes. The lack of Cdc25A destruction appears to be independent of ATM activity on account of the fact that Cdc25A is still present following high levels of DNA damage when ATM and Chk1 are active. However, the inability of DSBs to block Cdc25B activity seems to be ATM/Chk1-dependent since high levels of DNA damage cause a dramatic inhibitory phosphorylation of the phosphatase (Marangos and Carroll, 2012). Cdc25B inactivation could explain the sustained prophase arrest observed following significant levels of damage. This is not surprising considering that Cdc25B is irreplaceable in oocytes and the absence of Cdc25B, as in Cdc25B null mice, leads to female infertility due to the inability of oocytes to enter M-phase (Ferguson et al., 2005).

Besides DSBs, another type of highly toxic DNA lesions, interstrand crosslinks (ICLs), do not appear to activate an efficient DDR. In fully grown mouse oocytes, a major ICL repair factor, the Fanconi Anemia protein FANCD2 fails to be recruited to the sites of the DNA lesions (Yuen et al., 2012). Therefore, ICLs are not being repaired. Nevertheless, the oocytes enter M-phase without any delay. An explanation for these observations could be the possible absence of the activity of the ATM-related kinase, ATR. In somatic cells, ATR and its downstream effector Chk1 become active and enable a checkpoint in response to ICLs (Wang, 2007; Ben-Yehoyada et al., 2009). ATR is also required for the efficient monoubiquitination of FANCD2 enabling its role as an ICL repair factor (Andreassen et al., 2004). It would be interesting to see whether ATR can become active in fully grown oocytes in response to DNA damage. It is possible that, as in the case of ATM, ATR is either not expressed or unable to become recruited to the oocyte chromatin. This could explain the absence of FANCD2 and the subsequent inefficiency of the Fanconi Anemia pathway in fully grown oocytes.

It is not yet clear why fully grown oocytes cannot activate major DDR factors, such as ATM or repair factors, such as FANCD2. Although we have provided some possible explanations, further work is necessary in order to understand the mechanisms involved. Irrespective of how the system functions, it may be that oocytes have the capacity to resolve DNA lesions later in the cell cycle, perhaps during MI or MII, or even after fertilization during early embryonic development.

## MEIOTIC M-PHASE RESPONSE TO DNA DAMAGE

When a follicle reaches the pre-ovulatory stage it responds to the surge of LH and as a result the fully grown oocyte exits prophase arrest and enters MI. Resumption of meiosis leads to the first meiotic division and the extrusion of the first polar body (Pb1) which contains half of the homologous chromosomes and a minimum amount of cytoplasm. The oocyte then enters the second M-phase (MII) without an intervening interphase. It is at this stage the oocyte is ovulated and fertilization takes place. Egg activation triggers the completion of the second meiotic division and entry into the first embryonic cell cycle.

Considering the inability of meiotic prophase to establish a DDC, the two meiotic M-phases pose the only possible line of defense against DNA damage inflicted to the fully grown oocyte before the damage reaches the developing embryo. However, the knowledge on possible meiotic M-phase DDR mechanisms is extremely limited. When fully grown oocytes are exposed to the DSB-inducing agent Neocarzinostatin MI division is blocked (Yuen et al., 2012). It is not yet known, however, how sensitive this M-phase arrest is and which factors are implicated. Interestingly, the presence of ICLs in either MI or MII does not inhibit or delay cell division. However, ICLs formed in oocytes affect the quality and development of the resulting pre-implantation embryos (Yuen et al., 2012). Therefore, it seems that, the decision to establish or not an M-phase DDC depends on the type of DNA damage. More work needs to be done, in order to clarify the M-phase response to DNA damage.

## CONCLUDING REMARKS

The experimental evidence of especially the last decade has shed light into the diverse ways by which the mammalian oocyte responds to DNA damage. From our current knowledge we can assume that a DDC is necessary, primarily, for avoiding meiotic recombination errors in order to ensure correct chromosome segregation during the meiotic divisions. After birth, the TAp63-dependent checkpoint appears to be dispensable. The absence of apoptosis in damaged primordial follicle oocytes is not detrimental probably because the oocytes remain arrested at prophase where they have the time to repair any inflicted DNA damage. However, at the moment when a DDC is mostly needed, when the oocyte acquires the competence to enter M-phase, cell cycle arrest mechanisms that would respond to DNA damage are absent. It seems that the oocytes find preferable for DNA damage to be confronted later, in M-phase or the early embryonic cell cycles. Nonetheless, many important questions are still unanswered: is the recombination DDC a p63-dependent apoptosis mechanism? Why does the fully grown oocyte choose not to activate DDCs? What mechanisms are recruited in meiotic M-phase to respond to DNA damage? Therefore, there are still many pieces to be found in the puzzle that is the DDR of mammalian oocytes.

## Conflict of Interest Statement

The authors declare that the research was conducted in the absence of any commercial or financial relationships that could be construed as a potential conflict of interest.
